# Supplementary material for: Constructing and influencing perceived authenticity in science communication: Experimenting with narrative
Source: PLoS One. 2020 Jan 15;15(1):e0226711. doi: 10.1371/journal.pone.0226711 (PMC6961857; doi:10.1371/journal.pone.0226711)
Supplement: S1 Appendix — (DOCX) [file pone.0226711.s001.docx]

**Messages in each condition**

| **Condition** | **Message** |
| --- | --- |
| 1. Scientific brief written in conventional academic format (control) | The domestication of plants has been extremely important to human beings. Scientists want to learn more about when and where domestication first took place. Understanding where the first farmers lived and what they grew might help human beings produce food in the future. Until recently, scientists thought that the shift from the hunter-gatherer lifestyle to farming happened rapidly and in just a half dozen places on earth. In recent years, new technologies have led to new discoveries that have shown plant domestication to have been more widespread and older. For example, a new machine was used to determine the age of corn cobs found in ancient caves. It proved that corn was domesticated eight centuries earlier than previously thought. Discoveries like this have completely changed scientific understanding of when and where humans first domesticated plants. |
| 2. Scientific brief written in the first person | I can’t express enough how important the domesticating of plants has been to human beings. In my research, I want to learn more about when and where domestication first took place. I believe understanding where the first farmers lived and what they grew might help us produce food in the future. Until recently, we thought that the shift from the hunter-gatherer lifestyle to farming happened rapidly and in just a half dozen places on earth. In recent years, my research has been helped by some very important technological advances. For example, I used a new machine to determine the age of corn cobs found in ancient caves. I discovered that corn was domesticated eight centuries earlier than I had previously thought. This is the kind of evidence that has completely changed our understanding of when and where humans first domesticated plants. |
| 3. Scientific brief written in first person and referencing uncertainty in findings. | I can’t express enough how important the domesticating of plants has been to human beings. In my research, I want to learn more about when and where domestication first took place. I believe understanding where the first farmers lived and what they grew may help us produce food in the future. Until recently, we thought that the shift from the hunter-gatherer lifestyle to farming happened rapidly and in just a half dozen places on earth. In recent years, my research has been helped by some very important technological advances. For example, I used a new machine to determine the age of corn cobs found in ancient caves. I discovered that corn was domesticated eight centuries earlier than I had previously thought. This is the kind of evidence that has completely changed our understanding of when and where humans first domesticated plants. That being said, I can only study the remains of plants that we have found. If a plant didn’t leave any remains or if there are older unfound remains, I could be wrong about when humans first domesticated plants. |
| 4. Scientific brief written in first person and referenced the origin of the science communicator’s interest in the subject under study. | I can’t express enough how important the domesticating of plants has been to human beings. In my research, I want to learn more about when and where domestication first took place. I study plants now, but I first became interested in science when I was a child and wanted to travel in space and see alien life forms. Later, studying plants, I realized that they had all the variety and strangeness I had loved as a child. Studying plants was almost like studying aliens. I believe understanding where the first farmers lived and what they grew may also help us produce food in the future. Until recently, we thought that the shift from the hunter-gatherer lifestyle to farming happened rapidly and in just a half dozen places on earth. In recent years, my research has been helped by some very important technological advances. For example, I used a new machine to determine the age of corn cobs found in ancient caves. I discovered that corn was domesticated eight centuries earlier than I had previously thought. This is the kind of evidence that has completely changed our understanding of when and where humans first domesticated plants. |
| 5. Scientific brief written in first person and referenced earlier mistakes in the scientist’s interpretation of the data/analysis. | I can’t express enough how important the domesticating of plants has been to human beings. In my research, I want to learn more about when and where domestication first took place. I believe understanding where the first farmers lived and what they grew might help us produce food in the future. Until recently, we thought that the shift from the hunter-gatherer lifestyle to farming happened rapidly and in just a half dozen places on earth. But we were wrong about that. In recent years, my research has been helped by some very important technological advances. For example, I used a new machine to determine the age of corn cobs found in ancient caves. I discovered that corn was domesticated eight centuries earlier than I had previously thought. Some of these new results have revealed other mistakes that I made earlier in my analysis. For example, my earlier conclusions focused too much on human decisions and not enough on accidental events. New evidence has completely changed our understanding of when and where humans first domesticated plants. |

**Questionnaire**

[Text in square brackets did not appear in the actual survey]

**[Attention check]**

The passage you just read talks about_______?

- Bananas
- Dogs
- Corn cobs
- Chickens

**[Authenticity scale]**

How much do you agree with the statement: *This researcher is a human being first and a scientist second?*

Strongly disagree 1 – 2 – 3 – 4 – 5 Strongly agree

How likely is this researcher to share results that put their theories in doubt?

Least likely 1 – 2 – 3 – 4 – 5 Most likely

How likely is it that this researcher would be willing to conceal results for personal gain?

Least likely 1 – 2 – 3 – 4 – 5 Most likely

How likely is this researcher to hide their true thoughts, feelings and doubts behind their role as a researcher?

Least likely 1 – 2 – 3 – 4 – 5 Most likely

How likely is it that this researcher would be swayed in their research for personal gain?

Least likely 1 – 2 – 3 – 4 – 5 Most likely

How likely is it that this researcher would discard data that challenges their results?

Least likely 1 – 2 – 3 – 4 – 5 Most likely

How much do you agree with the statement:
*This researcher wants to know the truth, even if it contradicts what they thought they would find.*

Strongly disagree 1 – 2 – 3 – 4 – 5 Strongly agree

How likely is this researcher to have original insights into the subject matter?

Least likely 1 – 2 – 3 – 4 – 5 Most likely

How influenced do you think this researcher is by factors outside the study (e.g. funders, employers or colleagues)?

Least influenced 1 – 2 – 3 – 4 – 5 Most influenced

How well do you think this researcher understands his or her own biases, motivations and influences?

Not at all 1 – 2 – 3 – 4 – 5 Very well

How well do you think this researcher understands why he/she does the things he/she does?

Not at all 1 – 2 – 3 – 4 – 5 Very well

How persuaded are you that this researcher is confident in their own values and beliefs?

Least persuaded 1 – 2 – 3 – 4 – 5 Most persuaded

How passionate do you think this researcher is about their area of research?

Least passionate 1 – 2 – 3 – 4 – 5 Most passionate

How strongly does this researcher allow him or herself to be influenced by other people?

Not at all 1 – 2 – 3 – 4 – 5 Most likely

How knowledgeable do you think this researcher is about their area of study?

Least knowledgeable 1 – 2 – 3 – 4 – 5 Most knowledgeable

How much does this research matter in the real world?

Not at all 1 – 2 – 3 – 4 – 5 Very much

How respectful do you think this researcher is of their audience?

Least respectful 1 – 2 – 3 – 4 – 5 Most respectful

How important is it to this researcher that you understand their findings?

Least important 1 – 2 – 3 – 4 – 5 Most important

To what extent is this researcher the type of person who would use their role as a scientist to place themselves above other people?

Not at all 1 – 2 – 3 – 4 – 5 Most likely

In your view, how authentic is the scientist communicating these findings?

Least authentic 1 – 2 – 3 – 4 – 5 Most authentic

**[Demographics]**

What is your biological sex?

- Male
- Female

What is your age? (Please type in your age. Numbers only.) _________

What is your race?

- White
- Black or African American
- American Indian or Alaska Native
- Asian
- Native Hawaiian or Pacific Islander
- Other _________________

What is the highest level of school you have completed or the highest degree you have received?

- Less than high school degree
- High school degree or equivalent (e.g., GED)
- Some college but no degree
- Associate degree
- Bachelor degree
- Graduate degree

To what extent do you consider yourself a Democrat or Republican?

- Strong Democrat
- Democrat
- Lean Democrat
- No preference
- Lean Republican
- Republican
- Strong Republican
